# Allogeneic stem cell transplantation in adult patients with acute myeloid leukaemia and 17p abnormalities in first complete remission: a study from the Acute Leukemia Working Party (ALWP) of the European Society for Blood and Marrow Transplantation (EBMT)

**DOI:** 10.1186/s13045-017-0393-3

**Published:** 2017-01-18

**Authors:** Xavier Poiré, Myriam Labopin, Johan Maertens, Ibrahim Yakoub-Agha, Didier Blaise, Norbert Ifrah, Gérard Socié, Tobias Gedde-Dhal, Nicolaas Schaap, Jan J. Cornelissen, Stéphane Vigouroux, Jaime Sanz, Lucienne Michaux, Jordi Esteve, Mohamad Mohty, Arnon Nagler

**Affiliations:** 1 Section of Hematology, Department of Medicine, Cliniques Universitaires Saint-Luc, 10, avenue Hippocrate, 1200 Brussels, Belgium; 2 Acute Leukemia Working Party of the EBMT office, Paris, France; 3 Department of Hematology, University Hospital Gasthuisberg, Leuven, Belgium; 4 UAM allo-CSH, Hôpital HURIEZ, Lille, France; 5 Programme de Transplantation et Thérapie Cellulaire, Centre de Recherche en Cancérologie de Marseille, Institut Paoli Calmettes, Marseille, France; 6 Service des Maladies du Sang, CHRU, Angers, France; 7 Department of Hematology, Hôpital Saint-Louis, Paris, France; 8 Department of Medicine, Rikshospitalet, Oslo, Norway; 9 Department of Hematology, Radboud University Medical Center, Nijmegen, The Netherlands; 10 Daniel den Hoed Cancer Centre, Erasmus Medical Center, Rotterdam, The Netherlands; 11 Service d’Hématologie, CHU Bordeaux, Pessac, France; 12 Servicio de Hematologia, Hospital Universitario La Fe, Valencia, Spain; 13 Center for Human Genetics, KU Leuven and University Hospitals, Leuven, Belgium; 14 Hematology department, IDIBAPS, Hospital Clinic, Barcelona, Spain; 15 Service d’Hématologie clinique, Hôpital Saint-Antoine, AP-HP, Université Pierre et Marie Curie, INSERM UMRs U938, Paris, France; 16 Chaim Sheba Medical Center, Tel-Hashomer, Israel

**Keywords:** Acute myeloid leukaemia, 17p abnormalities, Stem cell transplantation, Survival, First remission

## Abstract

**Background:**

Acute myeloid leukaemia (AML) with 17p abnormalities (abn(17p)) carries a very poor prognosis due to high refractoriness to conventional chemotherapy, and allogeneic stem cell transplantation (allo-SCT) appears as the only potential curative option.

**Methods:**

To address outcomes after allo-SCT in patients with abn(17p), we retrospectively analysed de novo or secondary AML undergoing SCT between 2000 and 2013 from the EBMT registry.

**Results:**

One hundred thirty-nine patients with confirmed abn(17p) have been selected. At the time of transplant, one hundred twenty-five were in first remission (CR1). Median age was 54 years old. Abn(17p) was associated with a monosomal karyotype in 83% of patients, complex karyotype in 91%, monosomy 5 or 5q deletion (-5/5q-) in 55%, monosomy 7 (-7) in 39% and both -5/5q and -7 in 27%. Seventy-three patients (59%) had a reduced-intensity conditioning regimen. The 2-year overall survival (OS) and leukaemia-free survival (LFS) were 28 and 24%, respectively. The 2-year non-relapse mortality (NRM) was 15%, and 2-year relapse incidence (RI) was 61%. The cumulative incidence of grade II to IV acute graft-versus-host disease (GvHD) was 24% and that of chronic GvHD was 21%. In multivariate analysis, the presence of a -5/5q- in addition to abn(17p) was significantly and independently associated with worse OS, LFS and higher RI. Age and donor types did not correlate with outcome. Conditioning intensity was not statistically associated with OS, LFS and NRM when adjusted for patients’ age.

**Conclusions:**

In contrast to the dismal prognosis reported for AML patients harbouring abn(17p) undergoing conventional chemotherapy, allogeneic SCT provides responses in about 25% of those patients transplanted in CR1.

**Electronic supplementary material:**

The online version of this article (doi:10.1186/s13045-017-0393-3) contains supplementary material, which is available to authorized users.

## Background

Allogeneic stem cell transplantation (allo-SCT) is now a standard approach recommended for patients with high-risk acute myeloid leukaemia (AML) in remission [1, 2]. High-risk AML is mainly defined by the presence of determined poor-risk cytogenetic abnormalities at diagnosis together with specific mutational events [3–6]. In general, conventional post-remission high-dose chemotherapy is not capable to eradicate the initiating stem cell leukemic clone of high-risk AML, harbouring strong chemoresistance mechanisms [7], and only the potent graft-versus-leukaemia arising after allo-SCT may overcome the poor prognosis of these high-risk AML subtypes [8]. Indeed, several reports have confirmed the significant advantage of allo-SCT in high-risk AML, especially when performed early in the course of the disease [9–11]. Among the heterogeneous group of high-risk AML, prognosis can be further stratified based on specific genetic abnormalities, and the potential benefit of allo-SCT differs between these diverse AML subtypes [12–16]. While, it is still questionable if distinct genetic abnormalities with a known worse outcome like complex karyotype (CK) and monosomal karyotype (MK) AML will get the same benefit from allo-SCT [17].

*TP53* is located in 17p13 chromosomal region and is one of the major tumour suppressor genes, often inactivated by deletion and/or mutation in many tumours [18]. It has been described in 10 to 15% of AML patients, with an increased frequency in elderly patients and secondary AML [19]. *TP53* inactivation is associated in AML with a significantly lower response to intensive chemotherapy, translating into a poor outcome [20]. Although *TP53* mutations/deletions show a high correlation with complex karyotype in AML [21–23], *TP53* mutations and/or loss have emerged as a strong and independent prognostic marker of very poor outcomes regardless of associated cytogenetic abnormalities [24, 25]. Thus, long-term disease control is observed in less than 5% of the patients harbouring the *TP53* mutations with conventional chemotherapy [25, 26]. Molecular screening for *TP53* mutations is not routinely performed, and loss or disruption of 17p13 (17p abnormalities, abn(17p)) is usually identified by FISH analysis [27]. In this context, the potential capability of allo-SCT to overcome the dismal prognosis of abn(17p) AML is of great interest, scarcely explored until now. A first report from Mohr et al. described the outcome of 47 allografted patients and did not show a different outcome compared to non-transplanted patients, raising the hypothesis of a lack of sensitivity of this entity to the potential benefit of graft-versus-leukaemia effect [28]. This detrimental effect of abn(17p) on allo-SCT outcomes has been confirmed in another report with an event-free survival (EFS) of only 11% due to a very high incidence of relapse [17]. A recent report from Middeke et al. described 201 patients with abn(17p) AML transplanted during the past decade, showing an overall EFS of only 12%, with a slight better outcome among the 84 patients allografted in first complete remission (3-year EFS 18 vs 7%) *p* < 0.001) [29]. The purpose of the current study was to explore the potential role of early-phase allo-SCT in abn(17p) AML in the multicenter, registry context of EBMT, with the aim to identify specific subsets of patients who could benefit from the procedure.

## Methods

### Data collection and patient selection

The data on patients over 18 years of age with a diagnosis of de novo or secondary AML transplanted with a related or unrelated donor were available from the EBMT registry. The latter is a voluntary working group of more than 450 transplant centres reporting regularly on their transplant activity. Only patients having available cytogenetics and transplanted between 1 January 2000 and 31 December 2013 have been selected. Patients with second allo-SCT have been excluded as well as those receiving a haplo-identical transplantation. Audits are routinely performed to insure the quality of the reported data. All patients provided informed consent on the use of their data in retrospective studies. The Review Board of EBMT approved this study. We identified a dataset of 10,799 patients with 5495 patients displaying an abnormal karyotype. All cytogenetic abnormalities have been carefully reviewed by two physicians (Xavier Poiré and Lucienne Michaux). Most centres report conventional karyotype and a few report also FISH results. Cytogenetic results found in the registry are complete or often partial depending on the reporting center. Based on available data, we kept for further analysis only patients for whom data were sufficient to confirm the presence of abn(17p). Abn(17p) were defined as loss of 17p13 (*TP53* locus) such as monosomy 17, deletion (17p), isochromosome 17q (i(17q)), addition (17p) or other abnormalities that disrupt the 17p13 locus. Only one center reported a patient with *TP53* mutation. Those selected patients have been further categorised as CK, MK, presence of monosomy 7, presence of loss of 5q and/or presence of a inversion of chromosome 3 (inv(3)). CK has been defined as the presence of 3 or more cytogenetic abnormalities. MK has been defined as two or more autosomal monosomies or one autosomal monosomy in combination with at least one structural chromosomal abnormality. A total of 139 patients from 78 centres met the criteria and have been selected for further analysis.

Myeloablative conditioning (MAC) has been defined as a regimen including total body irradiation (TBI) of more than 8 Gy or a busulfan dose of more than 10 mg/kg. Reduced-intensity conditioning (RIC) includes intermediate doses of alkylating agents such as 8–10 mg/kg busulfan, 80–140 mg/m^2^ melphalan, 600–1200 mg/m^2^ cyclophosphamide or 5–10 mg/kg thiotepa, and/or low-dose TBI (<3 Gy). The following variables have been selected and included in the analysis: year of transplantation, age, gender, status at transplantation, time to diagnosis to complete remission, time to complete remission to allo-SCT, number of induction courses, type of conditioning regimen, in vivo T cell depletion, type of T cell depletion, cytomegalovirus (CMV) status of donor and recipient, donor type, source of stem cells, Karnofsky performance status at transplantation, engraftment, presence of acute and chronic graft-versus-host disease (GvHD), grade of acute GvHD, presence of CK, MK, monosomy 7, loss of 5q and/or inv(3), cause of death. HLA typing was determined at 10 loci (A, B, C, DRB1, DQB1) by high-resolution techniques, although not all the centres report complete data on HLA. All unrelated donors were defined as HLA matched (10/10) or mismatched at 1 locus (9/10). Additional data have been collected on the therapy of relapsing patients when available. HLA data on cord blood (CB) were not captured in this study. Methods and definitions were similar to other studies performed by the Acute Leukemia Working Party of the EBMT [30–32].

### Statistical analysis and endpoint definitions

Endpoints included leukaemia-free survival (LFS), relapse incidence (RI), non-relapse mortality (NRM), overall survival (OS), acute and chronic GVHD and GVHD-free/relapse-free survival (GRFS). All outcomes were measured from the time of stem cell infusion. LFS was defined as survival without relapse; patients alive without relapse were censored at the time of last contact. OS was based on death from any cause. NRM was defined as death without previous relapse. GRFS was defined as survival without grade 3–4 acute GVHD, extensive chronic GVHD, relapse or death. Surviving patients were censored at the time of last contact. The probabilities of OS and LFS were calculated by the Kaplan-Meier test, and those of acute and chronic GVHD, NRM, and relapse by the cumulative incidence estimator to accommodate competing risks. Results are expressed with a 95% confidence interval (CI). For NRM, relapse was the competing risk, and for relapse, the competing risk was NRM. For acute and chronic GVHD, death without the event and relapse were the competing risks.

For all prognostic analyses, continuous variables were categorised and the median was used as a cut-off point. A Cox proportional hazards model was used for multivariate regression. Factors associated with a *p* value less than 0.05 by univariate analysis were included in the model. Results were expressed as hazard ratio (HR) with 95% confidence interval

All tests were two-sided. The type 1 error rate was fixed at 0.05 for determination of factors associated with time to event outcomes. Statistical analyses were performed with SPSS 19 (SPSS Inc./IBM, Armonk, NY) and R 3.0.1 (R Development Core Team, Vienna, Austria) software packages.

## Results

### Patients’ characteristics

A total of 139 patients with abn(17p) have been identified. There were 125 patients transplanted in first complete remission (CR1), while only 14 patients were transplanted in second remission (CR2). Because of the small number of patients transplanted in CR2, further analysis has been focused on CR1 patients. A detailed table of the different abn(17p) is available as a Additional file 1: Table S1.

A total of 125 patients with abn(17p) transplanted in CR1 have been analysed in November 2015. The median follow-up of the cohort was 21 months (ranging 3–146 months). The median age at transplantation was 54 years old (ranging 18–69 years old). The median year of transplantation was 2009 (ranging 2000–2013). The median time from diagnosis to CR1 was 57 days (ranging 18–170 days), and median time from CR1 to transplantation was 82 days (ranging 11–286 days). For 81 patients, we had information about the number of induction courses to reach CR1. Fifty-two had just one course, 27 had 2 and 3 needed 3 rounds of chemotherapy. Most patients were de novo AML (85%) and only 19 patients corresponded to secondary AML, seven of them arising from an antecedent myelodysplastic syndrome. The majority of patients were male (57%) and were transplanted with a Karnofsky performance status of more than 90% (70%). A sibling donor was used in 48% and an unrelated donor in 43% (10/10: *N* = 23 (64%); 9/10: *N* = 13 (33%), missing: *N* = 36) whereas a cord blood was used in 10 patients. Source of stem cell was mostly peripheral blood (76%). CMV status was positive in 68% of the patients and also 68% of the donors. Fifty-one patients received a MAC and 74 patients a RIC. In patients less than 50 years old, only 14 patients (26.4%) received a RIC but this number increased up to 60 (83.3%) in patients over 50 years old (*p* < 10^−5^). Most frequent MAC were the combination of cyclophosphamide and busulfan (*N* = 19) followed by the association of total body irradiation (TBI) with cyclophosphamide (*N* = 14). Most RIC were fludarabine and TBI (*N* = 25) closely followed by fludarabine and busulfan (*N* = 24). Seven patients received the sequential FLAMSA-RIC approach [33]. In vivo T cell depletion has been used in 64 patients. Among those, 47 patients received anti-thymocyte globulin (ATG) and 17 patients alemtuzumab. Regarding associated cytogenetic categories, most patients carried also a CK (*N* = 98) or a MK (*N* = 86). An inv(3) was present in only 3 patients. Monosomy 7 (-7) was seen in 41 patients and a monosomy 5 or a loss of 5q (-5/5q-) in 58 patients. Both -7 and -5/5q- were present together in 28 patients. Table 1 summarises the patients’ characteristics.

**Table 1:** Patients’ characteristics (*N* = 125)

Median age at SCT (range)	53.6 years old (18–69)
Median follow-up (range)	21 months (3.3–146)
Interval between diagnosis and CR1 (range)	56.5 days (18–170)
Intervals from CR1 to SCT (range)	81.5 days (11–286)
Median year of SCT	2009 (2000–2013)
Secondary AML, *N* (%)	19 (15.2%)
CMV+ patient, *N* (%)	84 (68.3%)
CMV+ donor, *N* (%)	65 (68.3%)
Karnofsky >90% at SCT, *N* (%)	83 (70.3%)
Gender, *N* (%)	
Male	71 (57%)
Female	54 (43%)
Donor type, *N* (%)	
Sibling	60 (48%)
Unrelated	54 (43.2%)
Cord blood	10 (8%)
Source of SC, *N* (%)	
BM	19 (15.2%)
PB	95 (76%)
CB	10 (8%)
Conditioning regimen, *N* (%)	
MAC	51 (41%)
RIC	73 (59%)
In vivo T cell depletion, *N* (%)	64 (51%)
ATG	47 (38%)
Alemtuzumab	17 (14%)
Monosomal karyotype, *N* (%)	86 (82.7%)
Missing, *N*	21
Complex karyotype, *N* (%)	98 (90.7%)
Missing, *N*	17
Inv(3), *N* (%)	3 (2.9%)
Missing, *N*	20
-7, *N* (%)	41 (39%)
Missing, *N*	20
-5/5q-, *N* (%)	58 (55.2%)
Missing, *N*	20
Both -7 and -5/5q-, *N* (%)	28 (26.9%)

*Abbreviations*; *N* number, *CR1* first complete remission, *SCT* stem cell transplantation, *AML* acute myeloid leukaemia, *CMV* cytomegalovirus, *BM* bone marrow, *PB* peripheral blood, *CB* cord blood, *MAC* myeloablative conditioning, *RIC* reduced-intensity conditioning, *ATG* anti-thymocyte globulin

### Engraftment and graft-versus-host disease

**Fig. 1:**
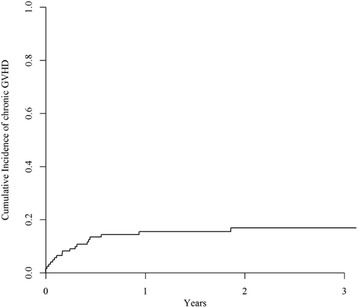
Cumulative incidence of chronic GvHD. The 2-year cumulative incidence of chronic GvHD was 21% [95% CI 14.2–29.5]

Engraftment was successful in 117 patients (94%). Six patients showed a graft failure and 1 patient lost his graft. The cumulative incidence of grade II to IV acute GvHD was 24%. The 2-year cumulative incidence of chronic GvHD was only 21% [95% CI 14.2–29.5] (Fig. 1). This low incidence is explained because many patients relapsed before developing chronic GvHD. A Cox proportional hazards model including age, donor type, use of ATG during the conditioning regimen, source of stem cells and conditioning intensity has been performed for cGvHD (Table 2). Only the use of ATG during the conditioning regimen was significantly associated with less chronic GvHD (HR 0.33, 95% CI 0.11–0.97; *p* = 0.04), whereas donor other than an HLA-matched sibling showed just a trend towards more chronic GvHD (*p* = 0.07).

**Table 2:** Multivariate analysis using a Cox proportional hazards model, *N* = 96. Chronic GvHD

	*p*	HR	95% CI	
Age ≥50 years old	0.41	1.51	0.56	4.06
Donor other than MSD	0.07	2.39	0.92	6.2
ATG vs No	0.04	0.33	0.11	0.97
PB vs BM	0.89	0.92	0.31	2.71
RIC vs MAC	0.12	2.34	0.8	6.86

*Abbreviations*: *MSD* matched sibling donor, *ATG* anti-thymocyte globulins, *PB* peripheral blood, *BM* bone marrow, *RIC* reduced-intensity conditioning, *MAC* myeloablative conditioning, *HR* hazard ratio, *CI* confidence interval

### Non-relapse mortality and relapse incidence

**Fig. 2:**
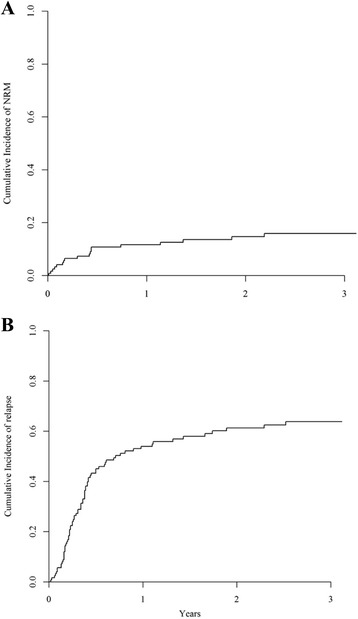
Non-relapse mortality (NRM) (**a**) and relapse incidence (RI) (**b**). The 2-year cumulative incidence of NRM was 15% [95% CI 8.9–21.8] (**a**) and the 2-year cumulative incidence of relapse was 61.3% [95% CI 51.5–69.7] (**b**)

The 2-year cumulative incidence of NRM was 15% [95% CI 8.9–21.8] as illustrated in Fig. 2a. None of the analysed variables (i.e. conditioning intensity, in vivo T cell depletion, use of ATG, age, donor or patient gender, female donor to male recipient, Karnofsky performance status, donor type, number of induction course, CK, MK, -7, -5/5q- or both -7 and -5/5q-) were significantly associated with NRM neither in univariate analysis nor in multivariate analysis.

Seventy-six patients relapsed at a median interval of 4 months from allo-SCT (range 0.2–92.8 months) translating into a 2-year cumulative incidence of relapse of 61.3% [95% CI 51.5–69.7] as illustrated in Fig. 2b. The 2 factors significantly associated with a higher RI were conditioning intensity and presence of -5/5q-. On the contrary, in vivo T cell depletion was not associated with higher relapse rate. The relapse incidence was 53% [95% CI 37–66.2] after a MAC and 68% [95% CI 55.2–78.2] when a RIC was used (*p* = 0.01). The presence of -5/5q- was associated with a significant higher RI (70% [95% CI 55.4–80.9]) compared to patients without -5/5q- (51% [95% CI 35.4–65.4], *p* = 0.03). Patients’ age above 50 years old and MK showed only a trend towards a higher RI (*p* = 0.06 and *p* = 0.05, respectively). The number of induction courses to reach CR1 did not impact on relapse rate. In multivariate analysis, only the presence of -5/5q- kept its significant impact on RI (*p* = 0.03) while conditioning intensity, age and MK did not show a significant impact on relapse risk (Table 3). Among the 56 relapsed patients with available information, 16 patients received donor leukocyte infusion (DLI). Thirteen of them received a second allo-SCT thereafter and 2 additional patients received a second allo-SCT as the only cell-based therapy for relapse. The 2-year probabilities of OS were 8.4% after DLI [95% CI 0–24.1] and 20% after second allo-SCT [95% CI 0–55.1].

**Table 3:** Multivariate analysis using a Cox proportional hazards model, *N* = 90. Only variables with *p* < 0.05 in univariate analysis. LFS, OS and RI

		*p*	HR	95% CI	
LFS	Age ≥50 years old	0.48	1.23	0.69	2.20
	RIC vs MAC	0.13	1.54	0.89	2.68
	MK	0.21	1.57	0.77	3.19
	Monosomy 5q	0.02	1.83	1.09	3.07
OS	Age ≥50 years old	0.35	1.37	0.71	2.64
	RIC vs MAC	0.07	1.75	0.95	3.24
	MK	0.14	1.79	0.82	3.91
	Monosomy 5q	0.01	2.02	1.18	3.47
RI	Age ≥50 years old	0.54	1.21	0.65	2.25
	RIC vs MAC	0.13	1.58	0.87	2.88
	Monosomy 5q	0.03	1.84	1.06	3.19

*Abbreviations*: *N* number, *LFS* leukaemia-free survival, *OS* overall survival, *RI* relapse incidence, *HR* hazard ratio, *CI* confidence interval, *MAC* myeloablative conditioning, *RIC* reduced-intensity conditioning, *MK* monosomal karyotype

### Survival

**Fig. 3:**
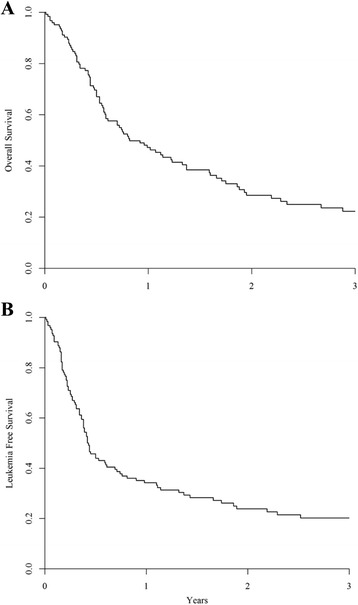
Overall survival (OS) (**a**) and leukaemia-free survival (LFS) (**b**). In the whole cohort, the 2-year probability of OS was 28% [95% CI 19.7–37.1] (**a**) and the 2-year probability of LFS was 24% [95% CI 15.7–31.9] (**b**)

**Fig. 4:**
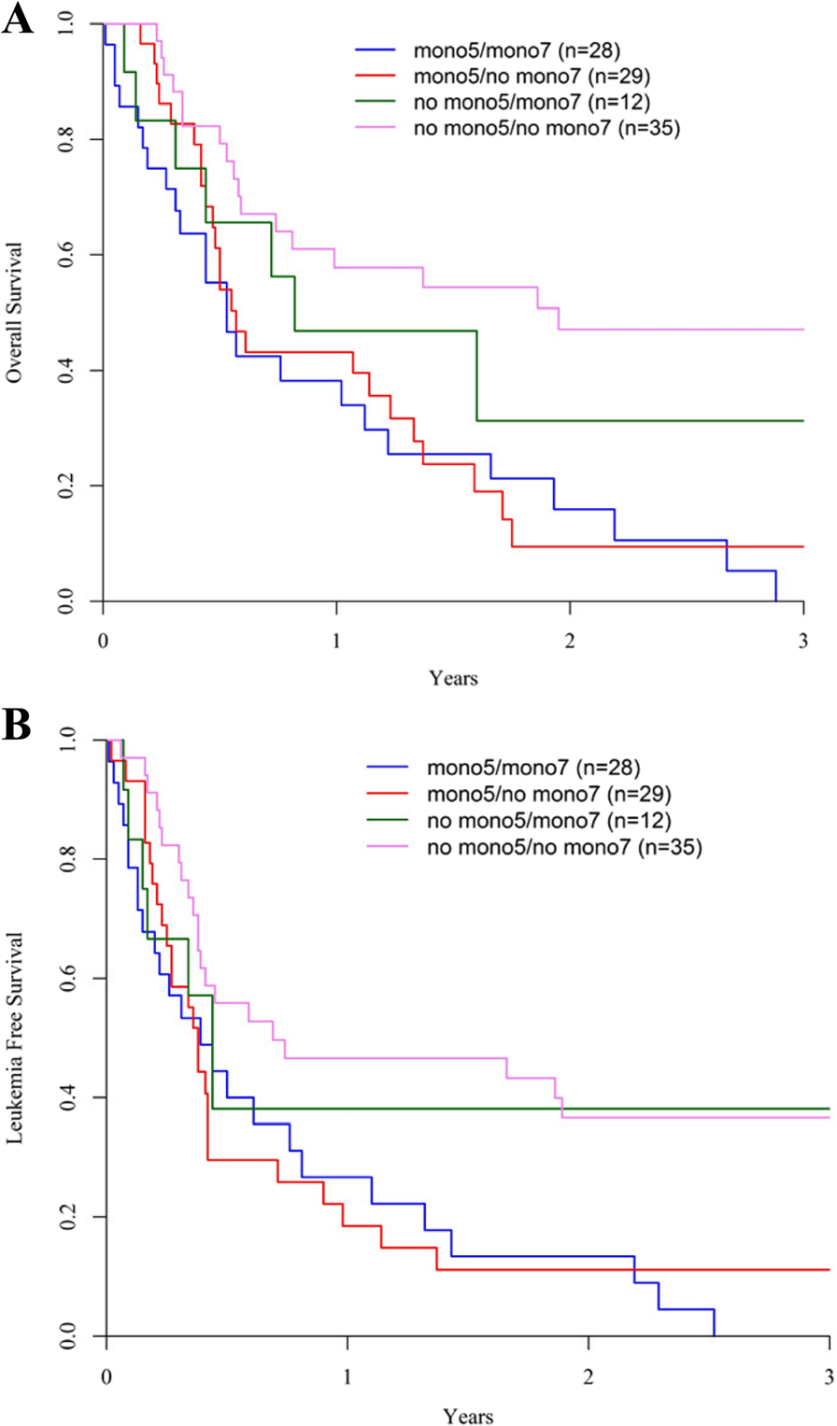
Overall survival (OS) (**a**) and leukaemia-free survival (LFS) (**b**) by cytogenetics subgroup. Mono5 refers as the presence of monosomy 5 or loss of 5q and mono7 refers as the presence of monosomy 7. Absence of Mono5 was associated to a better OS (2-year OS 47% [95% CI 30–65] and 31% [95% CI 0–63] in patients without and with mono7, respectively) compared to the subset of patients harbouring mono5 (2-year OS: 16% [95% CI 1–31] and 10% [95% CI 0–22], according to simultaneous mono7 or not, respectively) (**a**). The deleterious impact of mono5 on LFS was independent of presence of additional mono7, with a 2-year LFS of 11% [95% CI 0–23] (mono5 without mono7) and 13% [95% CI 0–27] (mono5 with mono7) vs 38% [95% CI 9–67] (absence of mono5 with mono7) and 37% [95% CI 20–53] (absence of both abnormalities, *p* = 0.007) (**b**)

The 2-year probability of OS was 28% [95% CI 19.7–37.1] (Fig. 3a). In univariate analysis, factors significantly associated with a worse OS were RIC, older age, MK and presence of -5/5q-. Monosomy 7 showed only a trend towards a decreased OS (*p* = 0.06 and *p* = 0.08, respectively). Thus, the 2-year probability of OS was 40% [95% CI 25–55] after a MAC and 21% after a RIC [95% CI 10–31] (*p* < 0.005). Patients above 50 years old had a worse OS (22%, [95% CI 12–36]) than younger patients (39%, [95% CI 23–54], *p* < 0.005). Given the strong interaction between use of RIC and older age, as previously described, conditioning intensity did not show any impact on OS when adjusted for age. Concerning associated cytogenetic categories, patients harbouring a MK had a decreased 2-year OS (19%, [95% CI 17–36]) compared to patients without this cytogenetic abnormality (58%, [95% CI 34–82], *p* = 0.005) as well as patient with concomitant -5/5q- (12%, [95% CI 2–22] vs 44%, [95% CI 28–59], *p* < 0.005). Taking together -7 and -5/5q- status, absence of -5/5q- was associated to a better outcome (2-year OS 47% [95% CI 30–65] and 31% [95% CI 0–63] in patients without and with concomitant -7, respectively) compared to the subset of patients harbouring -5/5q- (2-year OS 16% [95% CI 1–31] and 10% [95% CI 0–22], according to simultaneous -7 or not, respectively) (Fig. 4a). Thus, in multivariate analysis, the presence of -5/5q- was significantly associated with a decreased OS (HR 2.02; 95% CI 1.2–3.5, *p* = 0.01), whereas RIC was associated with a trend towards a worse OS (*p* = 0.07) (Table 3). Causes of death were disease related in 63 patients, infections in 13, GvHD in 11, haemorrhage in 1 and others in 2 patients.

The 2-year probability of LFS was 24% [95% CI 15.7–31.9] as illustrated in Fig. 3b. In univariate analysis, decreased LFS was significantly associated with RIC (2-year LFS of 20% [95% CI 8–29] and 30% [95% CI 16–44] after RIC and MAC allo-SCT, respectively, *p* = 0.01), age above 50 years old (2-year LFS: 20% [95% CI 10–30] vs 29% [95% CI 15–43], *p* < 0.005), MK (2-year LFS 17% [95% CI 8–25] vs 49% [95% CI 25–72], *p* = 0.02), and presence of -5/5q-). The deleterious impact of 5q loss was independent of presence of additional -7, with a 2-year LFS of 11% [95% CI 0–23] (-5/5q- without -7) and 13% [95% CI 0–27] (-5/5q- with -7) vs 38% [95% CI 9–67] (absence of -5/5q- with -7) and 37% [95% CI 20–53] (absence of both abnormalities, *p* = 0.007; Fig. 4b). In vivo T cell depletion and the use of ATG were not significantly associated with worse LFS. In multivariate analysis, only the presence of -5/5q- remained significantly associated with a decreased LFS (*p* = 0.02) (Table 3).

The presence of chronic GvHD was associated with significantly decreased risk of RI, but resulted in higher NRM, and worse OS and LFS (*p* < 0.005) (Table 4). The 2-year probability of GvHD and relapse-free survival (GRFS) was 16% [95% CI 9–23]. Among the 10 patients transplanted with CB, we found similar 2-year OS and LFS of 27% [95% CI 0–56] and 27% [95% CI 2–58], respectively. No significant differences were found with the other patients (*p* = 0.95 and *p* = 0.81, respectively). In this small cohort, four out of them showed the combination of abn(17p) and -5/5q-.

**Table 4:** Multivariate analysis using a Cox proportional hazards model. Impact of cGvHD on outcomes (time**-**dependant variable)

	*p*	HR	95% CI	
RI	<10^−4^	0.76	0.69	0.85
NRM	<10^−4^	2.92	2.62	3.25
LFS	<10^−4^	1.45	1.35	1.56
OS	<10^−4^	1.25	1.17	1.35

*Abbreviations*: *N* number, *LFS* leukaemia-free survival, *OS* overall survival, *RI* relapse incidence, *NRM* non-relapse mortality, *HR* hazard ratio, *CI* confidence interval

## Discussion

P53 loss of function resulting from chromosomal losses of 17p region and *TP53* gene mutations result in marked chemorefractoriness and very poor prognosis, with virtually incurability for most patients treated with conventional AML chemotherapy [24, 25, 28, 29]. The present study focused on the capability of allo-SCT to circumvent this dismal prognosis. In patients allografted in CR1, LFS at 2 years was 24%, suggesting the potential curability of a proportion of these patients with this approach, and the existence of a potent graft-versus-tumour effect capable to sustain response. Our cohort might correspond to a highly selected patient population, with some degree of chemosensitivity sufficient to achieve an initial response, and is therefore not representative of the whole abn(17p) AML. However, these results confirm the role of allo-SCT as a reasonable option for the subset of patients achieving sufficient cytoreduction at the time of transplantation. Abn(17p) are highly represented in overlapping cytogenetically very high-risk AML, such as MK and CK, and might participate in the underlying mechanisms responsible of the their refractoriness to standard intensive AML therapy. Nonetheless, the study provides evidences of the urgent unmet need to develop novel strategies for these patients. Different transplant modalities, concerning donor source or conditioning regimen, did not have a major impact on transplant outcome in our study, and future improvement attempts must explore pre- and post-transplant interventions, together with innovative modifications of allo-SCT conditioning regimen.

Our results are quite comparable to those reported by Middeke et al. [29]. The 2-year LFS and OS of 24 and 28%, respectively, in our cohort are more favourable compared to the previous retrospective study from Mohr et al., based on 47 transplanted patients, which did not show any advantage compared to conventional therapy, with a 4-year probability of survival for the entire cohort of only 4% [28]. Relapse was the main cause of treatment failure, achieving 70% at 2 years after RIC conditioning, and these relapses occurred at a median interval from transplant of 4 months, indicating the need of implementing early interventions in the post-transplant period to prevent relapse. Notably, the current results in patients with AML harbouring abn(17p) are similar to those observed with MK and CK AML. In those studies, an independent effect of abn(17p) has not been found [13–15]. In fact, genomic losses of 17p, together with losses of 5q and 7q, and gains 11q and 8q, are the most frequent cytogenetic abnormalities described in CK [34]. Nevertheless, our study cohort represents a more homogeneous population than the one addressed in the studies evaluating MK and CK AML. On the other hand, we were not able to find a significant effect of the presence of MK, probably because 83% of patients displayed MK at diagnosis.

NRM was only 17%, probably reflecting the positive selection effect in this population, enriched with responsive patients to previous chemotherapy. In fact, these 139 patients represent only 1.3% of 10,799 patients with an available karyotype in the EBMT database, a lower proportion than the expected rate of 5–10% in general AML population [19]. These 1.3% of patients refer only to the proportion of abn(17p) AML patients who were in remission and fit enough to survive until the transplantation procedure. Lower intensity conditioning regimens were associated to a higher relapse risk, up to 70%, in the univariate analysis, an association not confirmed in the multivariate analysis adjusted for other variable such as concomitant presence of 5q loss and age. Nonetheless, the effect of different regimens aimed to enhance antitumour effect without increasing toxicity must also be explored in the next future.

Presence of chronic GvHD, analysed as time-dependant variable, was independently associated with a lower relapse risk (HR 0.76), supporting the existence of a genuine and potent graft-versus-leukaemia. This antitumour effect of chronic GvHD, nonetheless, did not result in a neat benefit due to its association to a higher NRM translating into worse OS and LFS. Recognition of a potential graft-versus-leukaemia effect in abn(17p) AML would give the basis to develop strategies aimed to harness this alloimmune effect in the early post-transplant period, such as early withdrawal of immunosuppression or administration of prophylactic donor leukocyte infusion [35, 36]. Post-transplant administration azacytidine might contribute to stimulate the antitumour donor graft effect by enhancing the expression of tumour and minor histocompatibility antigens, with the theoretical added advantage of avoiding an increased GvHD rate by expansion of T regulatory cell population. Several studies have demonstrated feasibility of azacytidine during the post-transplant period, and the correlation of the expansion determined cytotoxic T cell subsets against tumour antigens with a lower relapse incidence, but the clinical benefit of such strategy should be further proven [37–40]. Other innovative donor cell strategies such as NK cell infusion or Cytokine-induced killer population, with a theoretical lower potential of GvHD induction, must be of high interest in this setting [41–43]. Anyhow, based on the very short median time to relapse, post-transplantation interventions should be given early as a prophylactic or maintenance strategy. Vosaroxin [44, 45] is a quinolone derivative reported to be *TP53* independent and shows some clinical benefit in combination with high-dose cytarabine in relapsed patients. Currently, it is completely unknown if this agent, administered prior to allo-SCT, might result into improved outcome after allo-SCT, but it might constitute a model to bring abn(17p) AML with better response to allo-SCT.

Additional chromosomal 5q loss conferred an even worse outcome in this cohort of patients, with an increased relapse risk and 2-year OS and LFS of only 10 and 11%, respectively, regardless the presence of concomitant monosomy 7. It was the only independent prognostic factor in this patient population. While the biological basis accounting for this combined deleterious effect is mostly unknown, *TP53* mutations have been frequently observed in association with loss of 5q, up to 80% of cases in some series, suggesting cooperation between *TP53* mutations and loss of putative tumour suppressor genes localised in 5q region [46–48]. Previous reports supported this hypothesis that multiple candidate genes localised on 5q cooperate with *TP53* mutations in the pathogenesis of myelodysplastic syndrome or AML [49–51]. Many genes on 5q have been proposed, but recently, haploinsufficiency of *ERG1* and *APC* in combination with the early acquisition of *TP53* mutations have emerged as a potential mechanism leading to the development of a leukemic clone resistant to apoptosis and with increased genomic instability [48]. This translates into chemoresistance and worse outcomes confirmed in our study with patients harbouring both abn(17p) and -5/5q-. In this subgroup of patients, the benefit of allo-SCT appears very limited and new therapeutic strategies are strongly warranted. On the contrary, patients with abn(17p) without -5/5q- showed a relative good outcome after allo-SCT with a 2-year probability of LFS of 37–38%.

## Conclusions

Allo-SCT arises as the best therapeutic option to improve survival in selected patients harbouring abn(17p) and achieving CR after frontline chemotherapy, especially in the absence of -5/5q-. Nonetheless, clinical benefit of allo-HCT remains very limited, followed by a high relapse incidence. Recognition of a potential graft-versus-leukaemia effect preventing relapse in some patients gives the rationale basis for the development of early chemotherapy-based or cell-based strategies to prevent relapse and therefore to increase the potential benefit of allo-SCT in these patients.

## Additional file

Additional file 1:
**Table S1.** (DOCX 13 kb)

## Abbreviations

-5/5q-: Monosomy 5 or loss of 5q-
-7: Monosomy 7
Abn(17p): 17p abnormalities
Allo-SCT: Allogeneic stem cell transplantation
AML: Acute myeloid leukaemia
ATG: Anti-thymocyte globulin
CB: Cord blood
CI: Confidence interval
CK: Complex caryotype
CMV: Cytomegalovirus
CR1: First complete remission
CR2: Second complete remission
DLI: Donor leukocyte infusion
EBMT: European Society of Blood and Marrow Transplantation
EFS: Event-free survival
GRFS: GvHD-free/relapse-free survival
GvHD: Graft-versus-host disease
HLA: Human leukocyte antigen
HR: Hazard ratio
Inv(3): Inversion of chromosome 3
LFS: Leukaemia-free survival
MAC: Myeloablative conditioning
MK: Monosomal karyotype
NRM: Non-relapse mortality
OS: Overall survival
RI: Relapse incidence
RIC: Reduced-intensity conditioning
TBI: Total body irradiation
