# Supplementary material for: Allogeneic stem cell transplantation in adult patients with acute myeloid leukaemia and 17p abnormalities in first complete remission: a study from the Acute Leukemia Working Party (ALWP) of the European Society for Blood and Marrow Transplantation (EBMT)
Source: J Hematol Oncol. 2017 Jan 18;10:20. doi: 10.1186/s13045-017-0393-3 (PMC5241968; doi:10.1186/s13045-017-0393-3)
Supplement: Additional file 1: — Table S1. (DOCX 13 kb) [file 13045_2017_393_MOESM1_ESM.docx]

Additional file 1: Table S1

| **Abn(17p)** | **N** |
| --- | --- |
| -17 | 46 |
| del(17p) | 32 |
| add(17p) | 21 |
| i(17)(q10) | 14 |
| Translocations involving *TP53* locus | 10 |
| Unknown (reported as abn(17p) without further informations) | 8 |
| der(17p) | 7 |
| dic(X;17)(q26;p12) | 2 |
| Inv(17) | 1 |
| *TP53* mutation | 1 |
